# Neurobiological Study of Fish Brains Gives Insights into the Nature of Gonadotropin-Releasing Hormone 1–3 Neurons

**DOI:** 10.3389/fendo.2013.00177

**Published:** 2013-11-19

**Authors:** Tomomi Karigo, Yoshitaka Oka

**Affiliations:** ^1^Department of Biological Sciences, Graduate School of Science, The University of Tokyo, Tokyo, Japan

**Keywords:** *gnrh1*, *gnrh2*, *gnrh3*, electrophysiology, evolution, morphology, behavior, GnRH

## Abstract

Accumulating evidence suggests that up to three different molecular species of GnRH peptides encoded by different paralogs of *gnrh* genes are expressed by anatomically distinct groups of GnRH neurons in the brain of one vertebrate species. They are called *gnrh1, gnrh2*, and *gnrh3*. Recent evidence from molecular, anatomical, and physiological experiments strongly suggests that each GnRH system functions differently. Here, we review recent advancement in the functional studies of the three different GnRH neuron systems, mainly focusing on the electrophysiological analysis of the GnRH-green fluorescent protein (GFP) transgenic animals. The introduction of GFP-transgenic animals for the electrophysiological analysis of GnRH neurons greatly advanced our knowledge on their anatomy and electrophysiology, especially of *gnrh1* neurons, which has long defied detailed electrophysiological analysis of single neurons because of their small size and scattered distribution. Based on the results of recent studies, we propose that different electrophysiological properties, especially the spontaneous patterns of electrical activities and their time-dependent changes, and the axonal projections characterize the different functions of GnRH1-3 neurons; GnRH1 neurons act as hypophysiotropic neuroendocrine regulators, and GnRH2 and GnRH3 neurons act as neuromodulators in wide areas of the brain.

## Introduction

**Figure 1:**
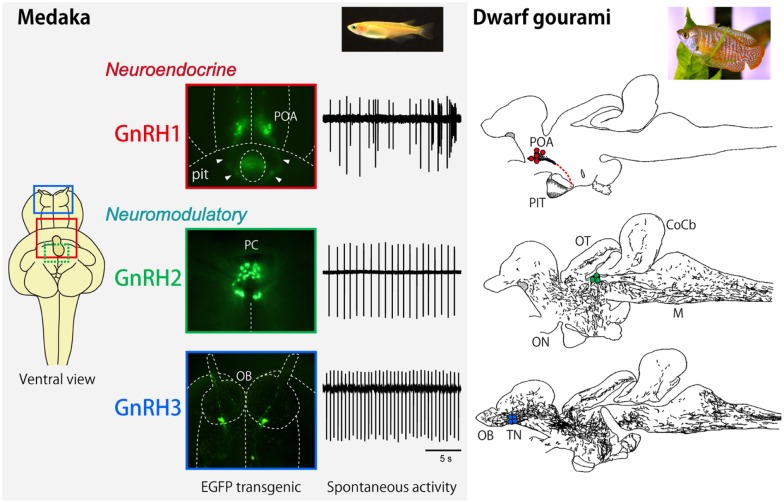
**Neuroanatomical and electrophysiological characteristics of GnRH1, GnRH2, and GnRH3 systems in medaka and dwarf gourami**. The inset for the left column indicates the ventral view of the GFP-transgenic medaka brain, showing the localization of GnRH1 (red), GnRH2 (green), and GnRH3 (blue) neurons. The GFP-labeled GnRH neurons and typical traces for their spontaneous electrical activities are shown for medaka. The right column illustrates the distribution of GnRH1 ∼3 immunoreactive cell bodies (colored dots) and fibers in sagittal brain sections. T, telencephalon; PIT, pituitary, CoCb, corpus cerebellum; ON, optic nerve; OT, optic tectum; M, medulla. The same abbreviations apply to Figure 5.

The conventional hypophysiotropic GnRH system (now referred to as GnRH1), which expresses *gnrh1* gene product, consists of neurons in the basal hypothalamic and/or preoptic area (POA) and project their axons to the median eminence or directly to the pituitary (in teleosts) and facilitates the release of gonadotropins from the pituitary. In addition to this hypothalamic GnRH system, there are two extrahypothalamic GnRH systems. The second one is called midbrain GnRH system (GnRH2), and the third one is called the terminal nerve (TN) GnRH3 system (Figure 1). The cell bodies that belong to the extrahypothalamic GnRH systems are located in the midbrain tegmentum (GnRH2) or the transitional area between the olfactory bulb and the telencephalon (GnRH3), and, in the both systems, the axons project widely throughout the brain but never to the pituitary. Therefore, it is clear that the two extrahypothalamic systems are not directly involved in the control of gonadotropin release from the pituitary, which is the main function of the hypothalamic/POA GnRH1 system (hypophysiotropic function). We have been suggesting that the TN-GnRH3 system, and probably the midbrain GnRH2 system as well, function as neuromodulators that regulate the excitability of other neurons in wide areas of the brain simultaneously (1–3).

The neurons of each group express different molecular species of GnRH peptide. The GnRH decapeptide of the hypophysiotropic GnRH1 neurons of mammals was first identified in the early seventies by two Nobel Prize winner groups (earlier called as LHRH; luteinizing hormone-releasing hormone). Since then, far more different molecular species of GnRH peptides have been identified. Owing to the development of synteny analyses of GnRH genes (4), it has been generally accepted that GnRH peptides are produced by either one of the three paralogous GnRH genes, *gnrh1* or *gnrh2* or *gnrh3*, which are considered to have originated as a result of two rounds of whole genome duplication events (5).

**Figure 2:**
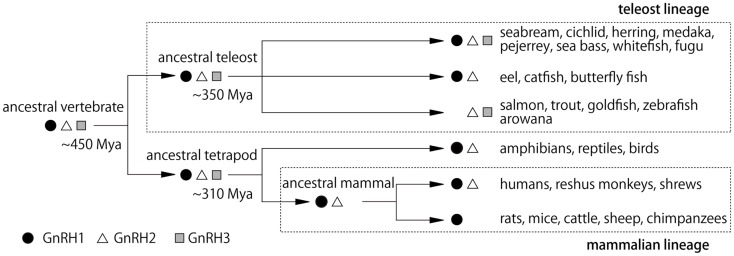
**Hypothetical scheme for the evolution of GnRH peptides in vertebrates**. Mya, million years ago (4).

It is noteworthy that all three GnRH systems are especially well developed in the teleost brains. Furthermore, it has been suggested that all three different paralogous genes for GnRH in the presumptive ancestral vertebrates are well preserved in many teleost brains, while one or two genes have been lost during evolution of vertebrates (Figure 2) (4). For example, the rodents and some mammals have lost both GnRH2 and 3, and primates and shrews are considered to have lost GnRH3. Accumulating evidence indicates that they have not actually lost the “functions” of these GnRH paralogs; instead of losing the functions of certain GnRH gene, there seem to be “functional compensations” by the remaining gene for the original functions of the lost genes. For example, *gnrh1* gene is lost in the zebrafish, but the hypophysiotropic function is considered to be performed by the *gnrh3*-expressing GnRH neurons in the POA. Conversely, eels and catfish appear to have lost *gnrh3* gene, but *gnrh1*-expressing neurons in the TN seem to compensate for the function of the GnRH3 system. On the other hand, all three paralogous genes, *gnrh1, gnrh2*, and gnrh3 are expressed and functional in the dwarf gourami (DG) and medaka, in which many results of neuroanatomical, electrophysiological, and behavioral experiments are available to date (1–3, 6–8). Thus, we will review recent advances in the neurobiological studies on the physiology and morphology of GnRH neurons, especially focusing on studies obtained in teleost brains.

## The Hypophysiotropic GnRH1 Neurons

The function of hypophysiotropic GnRH neurons (GnRH1 neurons) is well-conserved among vertebrates. The GnRH1 neurons stimulate gonadotropin secretion from the pituitary. In mammals, the GnRH1 neurons are located in the hypothalamic region and project their axons to the median eminence, and GnRH1 peptide is conveyed to the pituitary via the portal vessel. In the teleost fish, the hypothalamic GnRH1 neurons directly innervate the pituitary (9–11) and stimulate the gonadotropin secretion from the gonadotropes.

The early studies on the GnRH1 system were histological analysis and measurement of the amount of released hormones, gonadotropins, and GnRH, in the serum after intracerebroventricular applications or treatment of the hypothalamic explants or cultures with candidate regulatory factors upstream of GnRH1 neurons. Because of the technical limitations in those days, the intrinsic electrical properties of the GnRH1 neurons and their networks could not be well studied at the cellular level. The first report of electrophysiological recording of GnRH neurons from acute brain slice was performed in the genuine pig (12). This work and the other subsequent works (13–15) of Kelly and his colleagues revealed the intrinsic properties of mammalian GnRH1 neurons. In these studies, the electrical recordings from hypothalamic brain slices were followed by a *post hoc* identification of GnRH1 neurons by immunohistochemistry, and the percentage of successful recordings from the GnRH neurons was very low, because the GnRH1 neurons are scattered in the hypothalamus, and it was very difficult to identify GnRH neurons in the brain slice without any labeling. Cell physiological studies of GnRH1 neurons were greatly facilitated after the generation of transgenic mice expressing green fluorescent protein (GFP)-labeled GnRH1 neurons. Currently, several different kinds of transgenic animals labeled with GFP or calcium indicator are available for studying the physiology of GnRH1 neurons in mice, rats, and medaka (11, 16–21).

After the generation of transgenic animals, considerable numbers of electrophysiological analyses of GnRH1 neurons have been performed especially in mice and rats. By contrast, only two reports have been published concerning the non-mammalian GnRH1 neuronal activities; one in the cichlid fish (22) and the other in medaka (11). It should be noted that both the cichlid fish and medaka possess all three GnRH genes, and the GnRH1 neurons in POA project to the pituitary, and are thus clearly hypophysiotropic (9, 11). Especially in medaka, transgenic lines, in which three populations of GnRH neurons expressing either one of the three *gnrh* genes are specifically labeled with GFP, are already available and we can compare the properties of their electrical activities and other characteristics.

**Figure 3:**
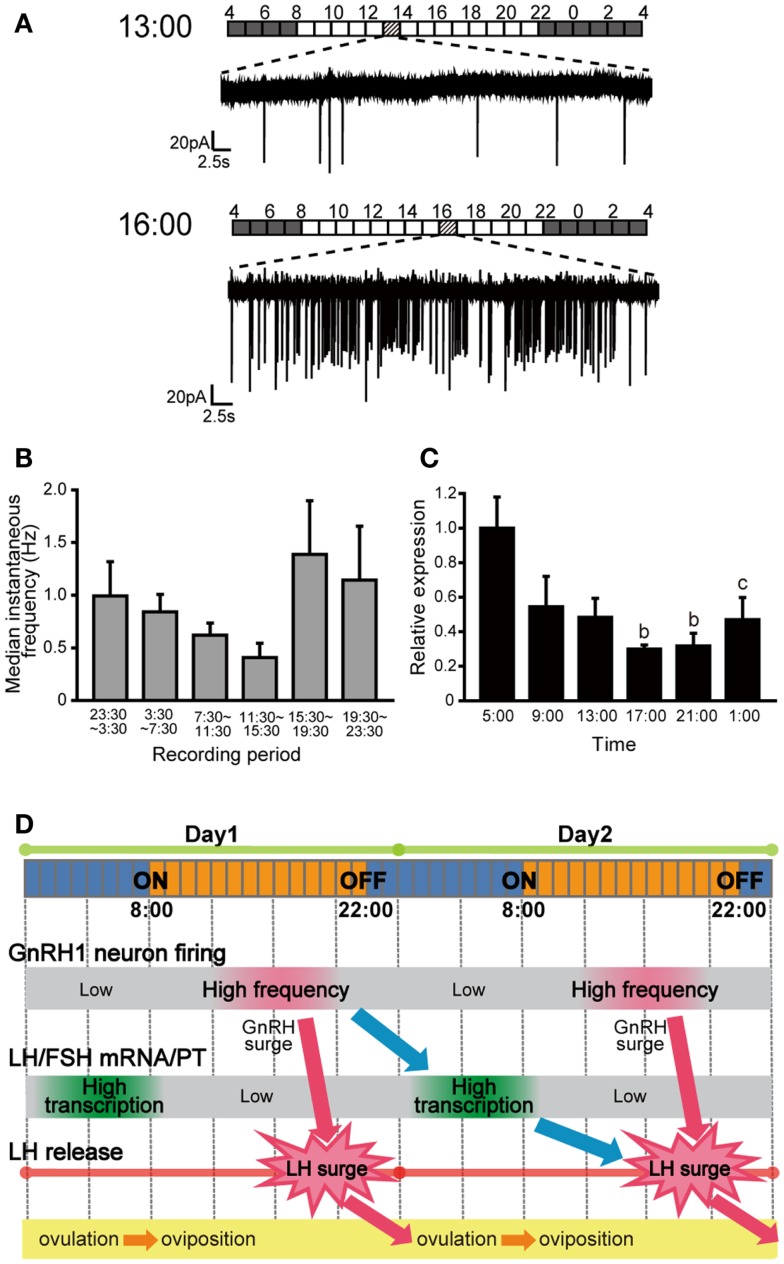
**Spontaneous electrical activity of GnRH1 neurons and the pituitary gonadotropin mRNA expression levels, and their time-of-day changes**. **(A)** Spontaneous firing activities of GnRH1-GFP neurons for 1 min in the time period preceding the putative LH surge (at 1300 h) and just before or during the putative LH surge (at 1600 h). Bars above the traces indicate the lighting conditions of the aquarium room. **(B)** Time-of-day changes in median instantaneous frequency (Hz) of the GnRH1-GFP neurons. **(C)** Time-of-day changes in pituitary *lhβ* mRNA expression levels. **(D)** Working hypothesis for the temporal regulation of the ovulatory cycle in the female medaka. The GnRH1 neuronal activity increases in the evening of Day 1, which causes the release of GnRH. GnRH stimulates release of LH from pituitary gonadotropes to cause the LH surge, which triggers ovulation during the night. GnRH1 peptide simultaneously acts on the pituitary gonadotropes to up-regulate *lhb* mRNA expression over the course of several hours, and LH thus synthesized and stored will be released during the LH surge on Day 2 (11).

In the medaka brain, there are two major populations of strongly GFP-positive GnRH1 neurons in the telencephalon: one in the dorsal group distributed in area ventralis pars dorsalis, supracommissuralis, and posterior of telencephalon [referred to as Vs/Vp in (23)] and the other in the ventral region ranging from the lateral part of area ventralis pars ventralis of telencephalon to the ventrolateral POA (vPOA) [referred to as Pbl and PPa in (23)]. From the retrograde labeling of the pituitary projecting neurons and semi-quantitative *in situ* hybridization, Karigo et al. (11) showed that vPOA GnRH1 neurons directly project to the pituitary and express much higher level of *gnrh1* mRNA than the dorsal GnRH1 neurons. Based on these results, they focused on the vPOA GnRH1 neurons as the typical hypophysiotropic population in medaka. By taking advantage of the small and transparent medaka brain and easy accessibility to vPOA population from the ventral side of the brain, spontaneous activity of vPOA GnRH1 neurons were recorded with loose-cell patch-clamp in a whole brain *in vitro* preparation, in which intact neural circuits are maintained. They showed irregular and episodic spontaneous firing activities, which is distinct from that of the extrahypothalamic GnRH2 and GnRH3 neurons in medaka (see below). The coefficient of variance (CV) of interspike interval, an index of the regularity of electrical activity, of the GnRH1 neuron spontaneous activity was much greater than those of GnRH2 and GnRH3 neurons in medaka (24). Similar irregular and episodic firing activities have been reported in the GnRH1 neurons of other vertebrates, such as mice, monkeys, and a cichlid fish (22, 25–27). Medaka is a long-day breeder and spawns daily under favorable breeding conditions, which means that they have 1 day estrous cycle. This feature makes medaka a nice model for the investigation of the relationship between the GnRH1 neuronal activities and the estrous cycle in gonadally intact condition. The spontaneous electrical activities of the vPOA GnRH1 neurons were recorded at various time-of-day, and they showed time-of-day dependent changes in their activities; firing activity in PM was significantly higher than that in AM in the reproductive females (Figures 3A,B). Because the firing frequencies of GnRH1 neurons have been suggested to be related to GnRH release (28–30), an increased level of GnRH release in PM is suggested. Also, the expression level of *lhβ* and *fshβ* mRNA showed changes related to the time-of-day, peaking early morning during the lights-off period (Figure 3C). In medaka, GnRH peptide application to the pituitary increase intracellular Ca^2+^ in LH cells within a few minutes (31). In contrast, transcription of *lhβ* mRNA increased several hours after GnRH application to the isolated whole pituitary culture. From these results, they proposed a working hypothesis concerning temporal regulation of the ovulatory cycle in the brain and pituitary (Figure 3D). This is the first report in vertebrate brains, in which time-of-day-dependent changes in the electrical activity of the GnRH1 neurons in the intact brain was recorded without manipulations of the steroid milieu.

Similar time-of-day dependent changes of GnRH1 neuronal activities have been studied in detail in ovariectomized and estrogen-implanted (OVX + E) mice model in a series of electrophysiological studies by Christian and Moenter (32). Normally, the intact female mice show an LH surge every 4–5 days, but the OVX + E mice show LH surge every afternoon. This model is convenient for analyzing the negative and positive feedback, because the mice show negative feedback state in the morning and positive feedback state in the evening in the same day. The spontaneous firing of GnRH1 neurons was low in the morning and high in the afternoon of OVX + E mice, but no such difference was apparent in OVX mice. The time-of-day-dependent changes in the firing activity in mice were thought to reflect an increase in GnRH release, which in turn triggers an LH surge. The results of their studies suggest that some kind of circadian neural mechanisms may be important for the regulation of GnRH neuronal activities (33).

There are two possibilities concerning the time-of-day-dependent changes in GnRH1 neuronal activities in general: changes in inputs to the GnRH1 neurons and the changes in the intrinsic excitability of the GnRH1 neurons themselves. In mice, the existence of both mechanisms has been suggested, which may exert synergistic effects on the neuronal activities of GnRH1 neurons (32, 34). Concerning the input to GnRH1 neurons, fast synaptic transmissions mediated by glutamate and γ-aminobutyric acid (GABA) exhibit time-of-day and estrogen-dependent changes, and are suggested to be important for the onset of LH surge. Not only glutamatergic but also GABAergic input via GABA_A_ receptor is excitatory for the GnRH1 neurons in mice and rats (35–38). Glutamatergic excitatory postsynaptic currents (EPSCs) to the GnRH1 neurons in OVX + E mice are decreased compared to those in OVX mice. This result suggests that the primary role of estradiol-dependent changes in glutamate transmission to GnRH1 neurons may be in mediating negative feedback (39). Importantly, GABAergic EPSCs to GnRH1 neurons showed time-of-day dependent changes exhibiting activities that are high in the afternoon and low in the morning only in OVX + E mice, not in OVX mice (40). This transition corresponds to the timing of positive feedback and negative feedback, and GABAergic transmission to GnRH1 neurons appears to be important for the onset of LH surge. Also, GnRH1 neurons are expected to receive inputs from the central circadian clock [reviewed in Ref. (41, 42)]. The GnRH1 neurons have been reported to receive monosynaptic inputs from the suprachiasmatic nuclei (SCN) (43–45), which is considered to be the master circadian clock in mammalian brain, and SCN lesion eliminates LH surge and ovulation (46). The clock gene mutant female mice show irregular estrous cycles and unorganized LH surge timing (47). In addition, neurons in the anteroventral periventricular nuclei (AVPV), which are directly upstream of GnRH1 neurons and are estrogen sensitive, also receive inputs from SCN (48). AVPV contains estrogen sensitive kisspeptin neurons, which are strong activators of GnRH1 neurons, and connection between SCN and AVPV kisspeptin neurons via vasopressin neurons was recently shown (49).

As for the intrinsic electrophysiological properties of the GnRH1 neurons, there are some lines of evidence to show that GnRH1 neurons themselves have some intrinsic properties that change according to the time-of-day. For example, the GnRH1 neurons have been reported to express endogenous clock genes (50), which may modify their own excitability by changing the expression levels of certain receptors or ion channels. For example, immortalized GnV-3 GnRH neurons express Gec1, a GABA_A_ receptor associated protein, and the expression level of *gec1* oscillates in a manner temporally related to the oscillations of the clock transcription factors (51). Also, high voltage-activated Ca^2+^ currents in the GnRH1 neurons were reported to vary with the time-of-day: currents in GnRH1 neurons recorded from OVX + E mice were smaller than those from OVX mice in the morning but were larger in the afternoon (52). These results are consistent with an increase of GnRH neuronal activity preceding LH surge (52).

The first report for recording from the non-mammalian GnRH1 neurons was made in the brain of a cichlid fish (*Astatotilapia burtoni*) (22). *A. burtoni* is known as a social fish, and the adult male becomes territorial (reproductively active) or non-territorial (reproductively regressed) according to their social environment. The GnRH1 neurons of the cichlid fish showed spontaneous and episodic firing activities, and there were no differences in the firing frequency between the territorial and non-territorial males. However, they found that the soma size from territorial males were larger than those from non-territorial males, and some basic electrical properties of the GnRH1 neurons were different between them accordingly; territorial GnRH1 neurons had higher membrane capacitance, lower input resistance, and shorter action potential, compared with those of non-territorial ones, which led to a tendency for neurons from non-territorial males to fire less rapidly in response to current injections. They proposed that these differences could serve to decrease GnRH release in non-territorial males. Although the social status of this fish is related to their reproductive state, this study implies that not only the reproductive state but also the social state may affect the regulation of GnRH1 neuronal activities.

## Regulators of GnRH1 Neuron Activities

The regulators of GnRH1 neuron activities are rather well studied in mammals [reviewed in Ref. (32, 34)], but are poorly understood in non-mammalian species. In teleost fish, most of the studies of the HPG axis regulation have been focused on gonadotropin secretion from the pituitary and the regulatory mechanisms of pituitary, and the number of neurobiological studies is quite small. However, some candidates have already been suggested as regulators of GnRH1 neuron activities, especially in the goldfish (53, 54). For example, dopamine inhibits the release of GnRH from brain slices including POA and the anterior hypothalamus (55, 56), and noradrenaline and serotonin stimulate the release of GnRH (57). Intraperitoneal injection of RFRP, a kind of RF-amide peptides first found in birds as an inhibitor of GnRH [reviewed in Ref. (58)], reduced the expression level of GnRH in the hypothalamus (59). NPY, which is implicated in the regulation of feeding in fish and mammals (60, 61) stimulates the release of GnRH from the GnRH terminals in the pituitary or in the brain slices containing POA (62). However, all of these experiments are pharmacological studies measuring the amount of GnRH1 peptide released into the serum after injection of drugs to the whole animal, or that released into the culture media or that remaining in the tissue after treating fragments of the pituitary or brain slices with drugs. Although these preceding studies may be suggestive for understanding the HPG axis regulation mechanisms in teleost fish, the detailed neuroendocrine regulatory mechanisms, for example, the actual action site of these candidates and the mode of actions on the GnRH1 neuronal activities are still controversial.

In the last decade, a peptide called kisspeptin, a hypothalamic peptide coded by *kiss1* gene, has been accumulating much attention as a powerful activator of GnRH1 neurons in mammals [reviewed in Ref. (63)]. In many mammals, it has been reported that the kisspeptin receptor, GPR54, is expressed on the GnRH1 neurons, and kisspeptin directly depolarizes the GnRH1 neurons and stimulate LH release from the pituitary. On the other hand, it has recently been found that the teleost fish possess two paralogous kisspeptin genes, *kiss1* and *kiss2*, and two subtypes of receptors, Gpr54-1 and Gpr54-2 [(64, 65); see (66) for our recent proposal for the nomenclature of kisspeptin receptors]. There have been some studies to show that intraperitoneal injection of kisspeptin increased the release or transcription of gonadotropins or GnRH1 in some fish species (67–70), it was earlier argued that kisspeptin also functions as a regulator of GnRH1 neurons in teleosts and GPR54 is also expressed in the GnRH1 neurons, as in mammals. However, recent studies using specific *in situ* hybridization for GPR54 report that the GnRH1 neurons do not express GPR54 in a cichlid, medaka, and sea bass (71–73), and kisspeptin does not increase the firing frequency of GnRH1 neurons in medaka in a loose-cell patch-clamp study (74). These recent results seem to suggest that kisspeptin(s) may not directly modulate GnRH1 neurons, in the same manner as in mammals, at least in some species of teleosts, and kisspeptin may have some functions other than HPG axis regulation.

In summary, the progress of the study of GnRH1 neurons as the regulator of the HPG axis in non-mammalian species, especially the cellular physiological study has been slow, compared to that of rodents, because of the technical limitations. However, the recent advances in the molecular genetic techniques are changing the situation of the studies in the non-mammalian species and in the non-model organism (also see below). Taken together with the study of rodent GnRH1 neurons, the study of non-mammalian GnRH1 neurons should facilitate our understanding of the basic biological functions of GnRH1 neurons in the central regulation of reproduction throughout vertebrates.

## The Midbrain GnRH2 Neurons

In contrast to the wealth of knowledge on the hypophysiotropic GnRH1 neurons, especially in mammals (see the previous section), and that on the extrahypothalamic GnRH3 neurons, especially in teleost fishes (see the next section), the study of GnRH2 neurons has been neglected, mainly because of the technical reasons. Although the GnRH2 peptides and the neurons expressing them are believed to be rather well-conserved among different vertebrate species (75), they have been lost during evolution in rodents (76). Therefore, there has been only a few studies on the behavioral functions of GnRH2 neurons, which has been performed mainly in the musk shrew (77–79). They suggested that the GnRH2 neurons are involved in the regulation of sex/feeding behavior dependent on the energy status. More recent studies on the goldfish similarly suggested feeding-related behavioral functions (80). However, the neural mechanisms of these suggested functions remain completely unknown, because there has thus far been no electrophysiological or other physiological analysis of the GnRH2 neurons at the cellular level.

Kanda et al. (24) recently established a GnRH2-GFP transgenic medaka, in which GFP expressing neuronal cell bodies were confined to the midbrain tegmentum just caudal to the crossing of the posterior commissure. Using such transgenic medaka, they demonstrated that most of the recorded GFP-GnRH2 neurons show regular spontaneous electrical activity (called “pacemaker activity” in the present paper; see the next section for details), which has already been shown to be characteristic of the TN-GnRH3 neurons (1, 2). The average spike frequency of the GnRH2 neurons was about one Hz, which was slightly slower than that of the GnRH3 neurons in medaka (1–2 Hz) (7) (also see below) or in the DG (about three Hz) (81–83). The CV, which is often used as an index of the regularity of electrical activity, for the interspike interval of the GnRH2 neuron spontaneous activity was about 0.3; the value is close to those of the GnRH3 neurons of the DG and medaka, which also show regular spontaneous pacemaker activities (Figure 1). Kanda et al. (24) also showed, by using whole cell current clamp recording, that the pacemaker activity of the GnRH2 neurons are not driven synaptically from other regular pacemaker neurons but are intrinsic to the GnRH2 neurons [see (24, 81) for detail].

Unfortunately, further physiological and behavioral analyses on the functions of GnRH2 neurons have not been published yet. Behavioral analysis of medaka after GnRH2 neuron-specific photo-ablation and gene knockouts are under way in our laboratory (Nishikawa et al., unpublished).

## The Terminal Nerve-GnRH3 Neurons

**Figure 4:**
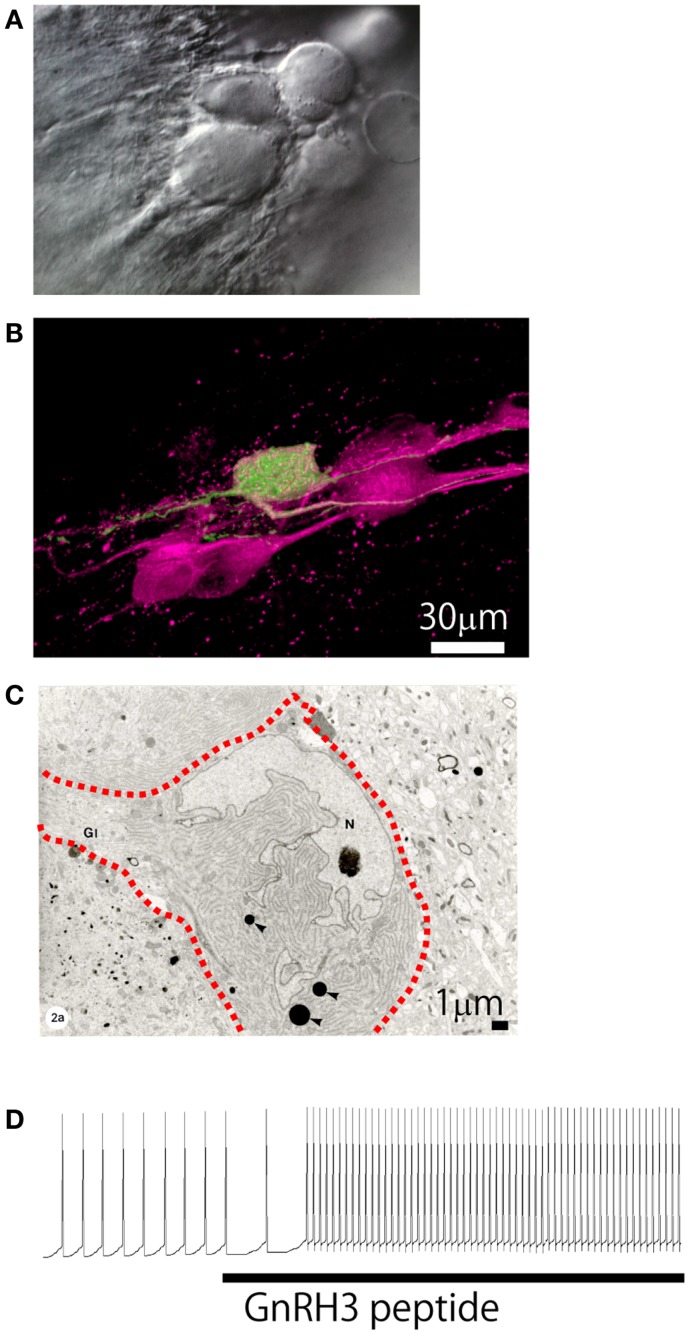
**Morphological and electrophysiological evidence for autocrine/paracrine regulation mechanism**. **(A)** Differential interference contrast micrograph of the cluster of TN-GnRH3 neurons. **(B)** Confocal micrograph of the cell cluster double-labeled for anti-sGnRH antiserum (magenta) and an intracellularly biocytin-labeled TN-GnRH neurons (green). Photo by Dr. H. Abe. **(C)** Electron micrograph of the cell cluster (three neurons; the interrupted red line indicates the cell border). **(D)** Electrophysiological recording showing upregulation of the pacemaker activity by the bath-applied GnRH3.

The neuroanatomical finding by Schwanzel-Fukuda and Silverman (84) of the rostral extrahypothalamic GnRH neurons, which was here identified as neurons belonging to the number zero cranial nerve, the TN, was really epoch-making for the study of extrahypothalamic GnRH neurons. After their report, the GnRH neurons in TN have been recognized in all vertebrate species reported to date, including humans (85). The function of the TN-GnRH3 system was originally suggested to be chemosensory (86). However electrophysiological studies later refuted this hypothesis (87). Instead, recent works mainly achieved in teleost model species indicate that the TN-GnRH3 system is most likely performing a neuromodulatory function. The earlier report by Demski and Northcutt (86) has, however, set the stage for the subsequent studies, which made many scientists to evaluate teleosts as really attractive animal models for the study of extrahypothalamic GnRH neurons. Later on, by taking advantage of the fact that the TN-GnRH3 neurons of a tropical fish (the DG) are especially large (approximately 20–40 μm or more in diameter) and form a conspicuous huge cell cluster in the ventral surface of the brain just beneath the meningeal membrane (Figure 4), Oka and Matsushima succeeded in recording electrical activity from a single GnRH neuron for the first time in vertebrate brains (81, 88). Here, they also took advantage of the fact that the whole brain of this fish can be maintained *in vitro* for a long period without oxygenation. This whole brain *in vitro* preparation of small fish (DG and medaka) has since been successfully used by Oka and his colleagues (1–3, 6), and Wayne and her colleagues (7, 8). GFP-GnRH transgenic zebrafish have also been developed recently for the electrophysiological study of GnRH neurons (89). Since no other neurobiological studies on physiology and morphology of TN-GnRH3 neurons are available in mammals or other non-mammalian species, we will mainly review results of these studies obtained in the teleost fish brains.

## Pacemaker Activity of TN-GnRH3 Neurons

As noted above, the electrophysiological recoding from a single TN-GnRH3 neuron was first reported by using the brain of a tropical fish, and the projection areas of a single neuron was also visualized by serial reconstruction of intracellularly labeled neurons (88). The authors found that the TN-GnRH3 neurons show spontaneous regular pacemaker activities of about three Hz, as described in the section above (THE MIDBRAIN GnRH2 NEURONS). The spontaneous regular pacemaker activity was clearly demonstrated to be dependent on the ion channels intrinsic to the TN-GnRH3 neurons. It was later shown by current clamp and voltage clamp electrophysiological studies (90, 91) that a tetrodotoxin-resistant persistent Na^+^ channels and some kind of counteracting K^+^ channels (92) are involved in the generation of such pacemaker activity. These studies took advantage of the conspicuous morphological features of the TN-GnRH3 neurons of a tropical fish, but Wayne and colleagues later reached similar conclusions about the intrinsic nature of the pacemaker potentials, by using a GFP-transgenic GnRH3 medaka (7). Although the identification of GnRH-immunoreactivity was lacking, similar pacemaker activity has also been reported in the older report in the carp and the goldfish (87, 93). Thus, it is suggested that the regular pacemaker activity of the TN-GnRH3 neurons is common to the teleosts, and probably the vertebrates in general.

## Regulatory Mechanisms of the Pacemaker Activity

### Excitatory autocrine/paracrine regulation

One of the most conspicuous morphological characteristics of the TN-GnRH3 neurons studied to date [teleosts and rodents such as rats, mice (94, 95), hamster (96), and guinea pig (84, 97)] is that the GnRH3 neurons make more or less tight cell clusters (Figures 4A,B). Oka and Ichikawa (98) reported on the ultrastructure of the TN-GnRH3 neurons in the DG (Figure 4C); the neighboring TN-GnRH3 neurons are in direct juxtaposition with one another without intervening glial cells. Furthermore, they found frequent occurrence of coated vesicles very close to the plasma membrane of the cell bodies and proximal dendrites, suggestive of membrane retrieval following active exocytosis, which most probably represent GnRH release from the somatodendritic area. The occurrence of spontaneous exocytotic release of GnRH peptides was actually demonstrated by the use of carbon fiber micro-amperometry [Ishizaki et al., unpublished; also see (28)]. It was also found that the TN-GnRH3 neurons express two different kinds of GnRH receptor genes (99). These lines of experimental evidence may suggest the occurrence of existence of electrical and/or chemical interactions among the GnRH3 neurons in the cluster (Figure 4, also see Figure 7) (2). In fact, electrophysiological evidence for the autocrine/paracrine regulation of the pacemaker frequency of the TN-GnRH3 neurons was reported (82, 100). The authors showed that extrinsic application of GnRH peptides to the perfusing solution, in which the pacemaker activity of the TN-GnRH3 neurons are recorded, increased the frequency of such activity (Figure 4D) (82). Functionally, the autocrine/paracrine regulation of the pacemaker frequency can facilitate “simultaneous activation of TN-GnRH3 neurons as a cluster,” once the pacemaker frequency of the TN-GnRH3 neurons are increased by synaptic inputs (see below) or other factors, the somatodendritic release of GnRH peptides will be facilitated (29), and the released GnRH peptides will up-regulate the pacemaker frequency of the GnRH3 neurons in the cluster (“positive feedback” mechanism).

### Synchronization via gap junctions

Besides this kind of simultaneous activation or positive feedback mechanism, the TN-GnRH3 neurons also show synchronization of pacemaker activity via gap junctions (101). These authors performed double patch-clamp current/voltage recordings of the TN-GnRH3 neurons and found that the simultaneously recorded pair of neurons fire action potentials synchronously with a small time delay, which is negligible compared to the interspike intervals of the pacemaker activity. Thus, the TN-GnRH3 neurons in the cluster form a functional assembly via electrical coupling, which results in synchronization of spontaneous regular firing activities. Furthermore, it has been suggested in the secretory processes of the endocrine and exocrine cells that the diffusion of metabolites through gap junctions may play an important role in signaling and synchronization (102). Therefore, in addition to the electrical coupling effects of gap junctional communications, we should also consider possible occurrence of metabolic coupling among the TN-GnRH3 neurons. We propose that these properties of the cluster of GnRH3 neurons plays a key role in the physiological functions of the neuromodulatory TN-GnRH system as described below in detail.

### Functional significance of TN-GnRH3 neuron clustering

**Figure 5:**
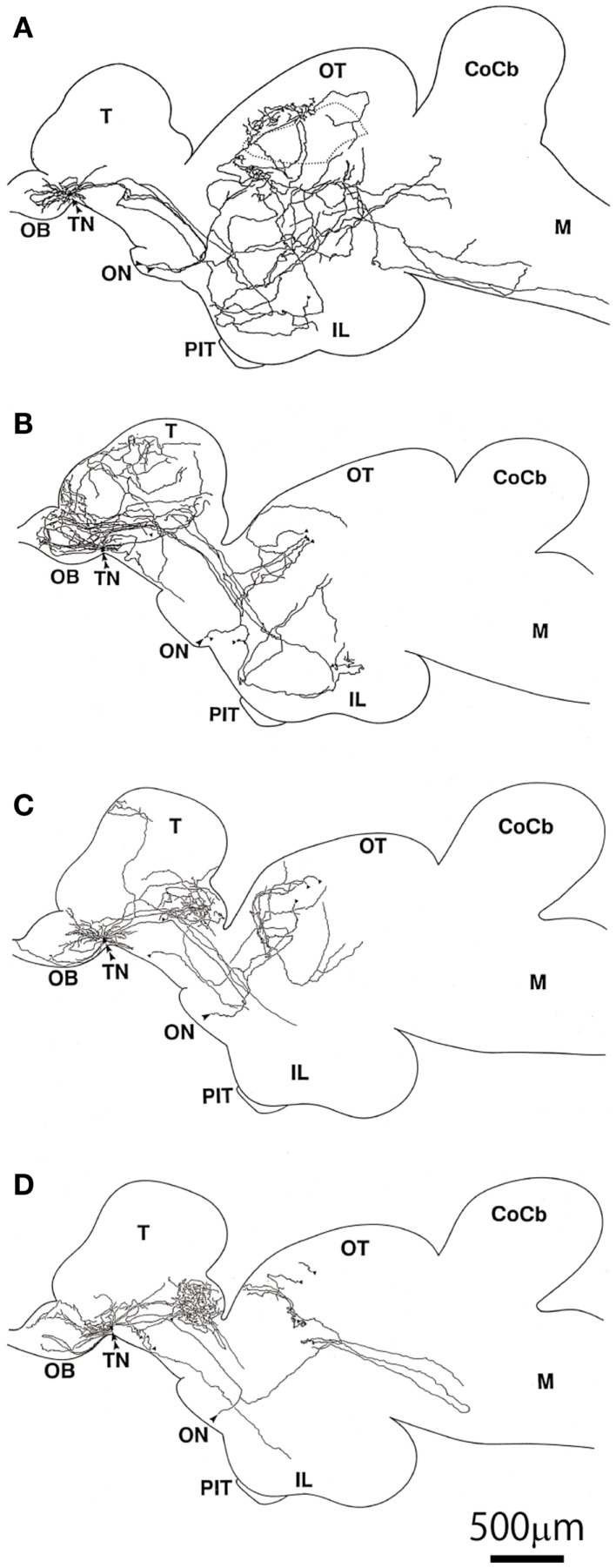
**Illustrations of intracellularly labeled TN-GnRH3 neurons reconstructed from serial sagittal sections**. **(A–D)** The double arrowheads indicate the cell bodies. The same scale bar applies to all illustrations (81).

A previous morphological study using intracellular labeling of single neurons (81) or extracellular labeling (103) demonstrated that each TN-GnRH3 neuron projects widely throughout the brain; the both studies reported that each neuron has a tendency to project more heavily to certain areas, one slightly different from another, but with considerable overlaps among the different neurons (Figure 5). Considering the fact that 10–20 TN-GnRH3 neurons on either side of the brain make cell cluster, the combined projection areas are considered to cover the entire brain, from the olfactory bulb to the rostral spinal cord, just like the distribution of GnRH3-immunoreactive fibers (104–107). Taken together with the electrophysiological demonstration of autocrine/paracrine positive feedback mechanisms, and the electrical and metabolic synchronizing mechanisms via gap junctions, and the morphological evidence shown here, we propose a working hypothesis that the clustering of the TN-GnRH3 neurons functions as an averaging device for the individual neuronal activities, which leads to the transmission of a coordinated uniform output, i.e., synchronized regular firing activity at certain frequency, throughout the projection areas covering wide areas of the brain simultaneously.

### Synaptic inputs to TN-GnRH3 neurons

In addition to the mechanisms intrinsic to the cluster of TN-GnRH3 neurons, there are extrinsic synaptic inputs that are either excitatory or inhibitory. Although the fine structural study revealed very few direct axo-somatic synapses, frequent occurrence of synaptic inputs have been identified in a special neuropil, which are formed by fine somatic processes or small dendrites (98). Electrophysiological studies have demonstrated glutamatergic excitatory as well as inhibitory synapses mediated by several different types of ionotropic and metabotropic glutamatergic receptors (108). More recently, excitatory GABAergic synaptic inputs via GABA_A_ receptors (109) have also been suggested, which is dependent on the higher intracellular Cl^−^ concentration of the TN-GnRH3 neurons. This kind of excitatory GABAergic synaptic inputs are also known to be characteristic of GnRH1 neurons of mice and rats (38). Patch-clamp recording of the whole cell current clamp or the less invasive loose patch on-cell recording of these neurons have actually shown that Glu and GABA can modulate the frequency of pacemaker potentials in either excitatory or inhibitory manners, depending on the type of receptors.

### Inhibitory peptidergic autocrine/paracrine and synaptic regulations

**Figure 6:**
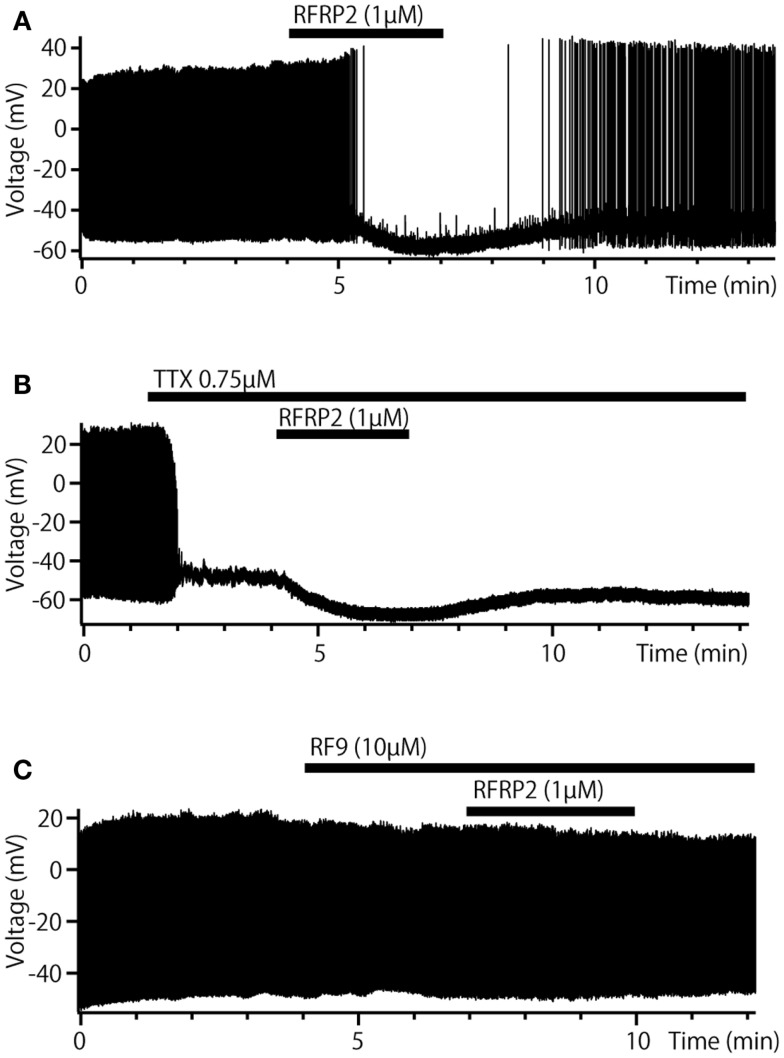
**Inhibitory synaptic regulation by RFRP of TN-GnRH3 neurons in dwarf gourami**. **(A)** Bath-applied RFRP2 reversibly decreases the frequency of pacemaker activity. **(B)** In the presence of TTX, RFRP2 hyperpolarizes the cell. **(C)** In the presence of RF9 (a potent antagonist of GPR147/74, candidate NPFF, and RFRP receptors) the inhibitory effect of RFRP2 is diminished.

Interestingly, recent physiological and anatomical studies revealed “inhibitory” (negative feedback) peptidergic autocrine/paracrine regulations of pacemaker potentials of the TN-GnRH3 neurons as well. It has been previously reported that the TN-GnRH3 neurons of the DG express FMRF-amide immunoreactive peptides (110), a molluscan cardioexcitatory peptides. It has recently been demonstrated that the peptide is actually NPFF, a peptide belonging to a big family of RF-amide peptides (111). The authors performed cloning and sequence analysis of the PQRF-amide (NPFF/NPAF) gene in the DG and found evidence to suggest that FMRF-amide-like peptide in TN-GnRH3 neurons of the DG is actually NPFF. They further studied electrophysiological effects of FMRF-amide and NPFF on the pacemaker potential of the TN-GnRH3 neurons and found that they inhibit the pacemaker activity of TN-GnRH3 neurons via G-protein coupled receptors, and this inhibition was diminished by RF9, a potent antagonist for mammalian NPFF receptors. Because the hyperpolarizing effect of these peptides remained after shutting off synaptic inputs with TTX, it was concluded that NPFF directly exerts its effect on the TN-GnRH3 neurons, which are considered to co-express NPFF peptides simultaneously with GnRH3. The expression of NPFF receptors (GPR74-1) in the TN-GnRH3 neurons of medaka was also demonstrated by *in situ* hybridization study (Yamamoto et al., unpublished). It was demonstrated by voltage clamp study that changes in the K^+^ permeability underlies the hyperpolarizing effect of NPFF (111). These results suggest that the TN-GnRH3 neurons possess autocrine/paracrine regulatory mechanisms of both facilitatory (positive feedback) and inhibitory (negative feedback) natures as well as a synchronizing mechanism via gap junctions (see below). Because the release of peptides is believed to be facilitated by high-frequency firing (see below), it is conceivable that the threshold for NPFF release may be higher than that for GnRH, and the NPFF inhibitory mechanism may function to stop or down-regulate the “overheated” pacemaker activity. Furthermore, a novel powerful inhibitory peptidergic synaptic inputs have been recently identified in the DG (83) as well as in medaka (Kanda et al., unpublished). Arg-Phe-amide (RF-amide) has been known as a novel family of peptides that are involved in various kinds of physiological regulation of autonomic functions including reproduction. Among the RF-amides, the RF-amide-related peptide (RFRP) neurons attract much attention, because RFRP has originally been found as a negative regulators of gonadotropin (FSH and LH) release (112–114), and it has also been sown to have inhibitory effects on the hypophysiotropic GnRH1 neurons (115). Moreover, in medaka, a neuroanatomical evidence demonstrated that RFRP neurons in the hypothalamus have robust projections to the vicinity of TN-GnRH3 neurons (116). The metastin (=kisspeptin)-immunoreactive neurons and fibers described in their report were recently identified as RFRP immunoreactive. Thus, it follows that TN-GnRH3 neurons receive robust projections from the hypothalamic RFRP neurons. It was demonstrated electrophysiologically that RFRP inhibit the pacemaker activity of the TN-GnRH3 neurons just like NPFF does so (Figure 6) (83). As shown in NPFF (see above), RFRP decreased the frequency of pacemaker activity (Figure 6A), hyperpolarized the GnRH3 neurons in the presence of TTX (Figure 6B), and the inhibition was diminished by RF9 (Figure 6C). The authors demonstrated that RFRP2 inhibit the pacemaker activity of TN-GnRH neurons by closing TRPC channels and opening voltage-independent K^+^ channels.

### Functional considerations for the regulation of spontaneous activities

**Figure 7:**
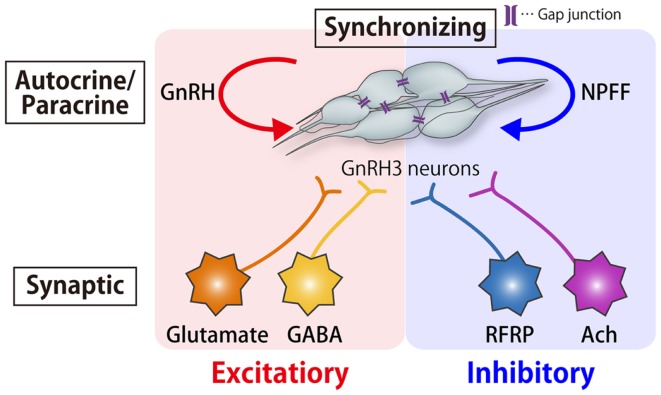
**Schematic drawing of the autocrine/paracrine, synchronizing, and synaptic mechanisms for the modulation of pacemaker frequency of the TN-GnRH3 neurons**. The tight cluster of TN-GnRH3 neurons release both GnRH and NPFF peptides, which function as autocrine/paracrine positive and negative feedback modulators, respectively. There is also a synchronizing mechanism for the pacemaker firings via gap junctions. In addition, there are excitatory (glutamatergic and GABAergic) and inhibitory (RFRPergic and AChergic) synaptic mechanisms for modulation of pacemaker activity.

Thus, the frequency of pacemaker activity of the TN-GnRH3 neurons is regulated by amazingly diverse mechanisms: peptidergic excitatory and inhibitory autocrine/paracrine mechanisms (GnRH and NPFF, respectively), synchronizing mechanism via gap junctions, and excitatory (via Glu and GABA) and inhibitory (via RFRP) synaptic mechanisms (Figure 7). Why are there so many mechanisms for the regulation of pacemaker frequency? One of the possible explanations may be that it is important for the proposed neuromodulatory functions of the TN-GnRH3 neurons. Although we do not have an answer to this question, there are a few lines of behavioral evidence to suggest the importance of pacemaker frequency for the neuromodulatory function of the TN-GnRH3 neurons. Ramakrishnan and Wayne (8) reported that social cues from conspecific medaka alter electrical activity of the TN-GnRH3 neurons via visual signals. One-day exposure of female medaka to visual and chemosensory cues of conspecific male fish suppressed the pacemaker frequency of female GnRH3 neurons compared with exposure to other females. They further noted that chemosensory cues alone were insufficient to induce this social cue response, but visual cues alone replicated the combined social cue response. Thus, they hypothesized that sensory signals, especially visual social cues, modulate the pacemaker frequency of TN-GnRH3 neurons. In fact, it has been reported anatomically that the TN-GnRH3 neurons receive strong synaptic inputs related to somatosensory and visual information from a tegmental nucleus and those related to olfactory information from the olfactory bulb and some telencephalic areas (117). Another recent behavioral study in medaka also stresses the importance of the frequency of pacemaker activity of the TN-GnRH3 neurons (Okuyama et al., submitted).

Another behavioral study, in which the TN-GnRH3 neuron clusters of both sides of the brain of the DG were electrolytically lesioned, suggested that the TN-GnRH3 neurons are involved in the control of motivational state of the animal (118). They compared the sexual behaviors before and after such lesions and focused on the frequency of nest-building behavior of the male fish. The male fish, control and experimental, were paired with a familiar partner female, and the frequency of nest-building was counted for 60 min. The characteristic behavioral change was the higher occurrence of failure of nest-building behavior of the male in the TN-lesioned fish. The behavior of the lesioned male fish was quite normal in the general sensory and motor aspects, and some percentage of males still could perform nest-building behavior when the female partner’s approaching and thrusting behaviors were strong enough. The authors proposed that the TN-GnRH3 neurons are involved in facilitation of the motivational state of the animal, and the lesions disrupt it. In mammals, there is only one similar behavioral result that the TN damage impaired the reproductive behavior of the male hamster (119).

Here we discuss the functional relationships between the regulation of the spontaneous activities (pacemaker frequency or pattern of spontaneous firing) of the TN-GnRH3 neurons and their neuromodulatory functions. It has been generally assumed that high-frequency firing, especially the burst firing, promotes peptide release (29, 120, 121). However, as described above, the TN-GnRH3 neurons usually fire at rather slow rates (1–6 Hz, average of about three Hz in the DG) and show spontaneous burst firing activities only infrequently (81). Thus, it remained to be elucidated whether the TN-GnRH3 neurons show burst activities, and if so, how the switching between the regular pacemaking and bursting modes is regulated in these neurons. Kawai et al. (122) recently found in the goldfish that a single pulse electrical stimulation of the neuropil surrounding the cluster of TN-GnRH3 neurons induce transient burst activities in TN-GnRH3 neurons. The authors suggested from their physiological and morphological data that this phenomenon occurs following long slow IPSPs mediated by cholinergic synaptic terminals surrounding the TN-GnRH3 neurons. This long IPSP induces sustained rebound depolarization that was suggested to be generated by a combination of persistent voltage gated Na^+^ channels and low-voltage-activated Ca^2+^ channels. Thus, the TN-GnRH3 neurons can be induced to fire in high-frequency burst mode by such synaptic activation, and such burst firing should contribute to the release of GnRH peptides. The released GnRH peptides are suggested to modulate neural functions in wide areas of the brain; e.g., neuromodulatory effect of GnRH on the synaptic transmission of the olfactory bulbar neural circuit has been reported in goldfish (123). But what about the physiological functions of the slow rate pacemaker activities? Akazome et al., recently suggested from their molecular biological data that the TN-GnRH3 neurons also express glutamate as a cotransmitters (124). It has been suggested in rats that hypophysiotrophic GnRH1 neurons also express glutamate as a co-transmitter (125). Therefore, it may be possible that the use of glutamate as a co-transmitter is a feature common to the GnRH neurons in general. Although there has not been any experimental evidence to suggest occurrence *in vivo* of glutamatergic conventional fast neurotransmission by the GnRH neurons, it is an attractive working hypothesis to assume that the TN-GnRH3 neurons perform glutamatergic neurotransmission during the low frequency pacemaker activity, and release GnRH peptides during high-frequency rebound burst firing activity after cholinergic slow and long hyperpolarization (122).

## Conclusion

As described in the present article, the introduction of GFP-transgenic animals for the electrophysiological analysis of GnRH neurons has greatly advanced our knowledge on their anatomy and electrophysiology. The hypophysiotropic hypothalamic/POA GnRH1 neurons of the rodents and medaka, both of which serve as good animal models for their physiological analyses, show irregular episodic spontaneous activity (Figure 1). The important physiological characteristic of the GnRH1 neurons is that the average frequency of their spontaneous activity show time-of-day dependent changes. Since the GnRH1 neurons directly project to the pituitary or the median eminence and release GnRH1 peptide to stimulate the pituitary gonadotrophs, such cyclic changes in the electrical activity can be considered to underlie the cyclicity of such regulatory mechanisms of reproductive functions.

The GnRH2 and GnRH3 neurons are so-called extrahypothalamic GnRH neurons and do not project their axons to the pituitary gland but to wide areas in the brain, and are therefore considered as neuromodulatory in nature. Both types of neurons show regular pacemaker activities that arise from the nature of their intrinsic ion channels. As detailed in various studies of the TN-GnRH3 neurons of teleosts, the morphological and physiological characteristics of the neuromodulatory GnRH neurons are appropriate for the simultaneous modulation of wide areas of the brain according to the synchronized and/or coordinated pacemaker frequency of the clustered population of GnRH neurons, which may act as an averaging device reflecting the physiological status of the animal.

Although the GFP-transgenic animals have been really helpful for the morphological and physiological studies of the GnRH1 ∼3 neurons, the technical applications of transgenic technology is not at all limited to the use of GFP-transgenic animals. For example, transgenic animals expressing calcium indicator proteins such as pericam or inverse pericam has been used for recording activities of GnRH neurons without the use of electrophysiology (126) or for recording the release activity of pituitary gonadotropins in response to GnRH stimulation (31). Another line of new transgenic technology includes the optogenetic activation of specifically gene-targeted neurons (127), which should prove to be useful for controlling activities of various types of GnRH neurons to analyze the physiological and behavioral effects of changing electrical activities of certain types of GnRH neurons. Also, new gene knockout techniques, such as TALEN (128) or CRISPR/Cas (129), are being developed at enormously fast speed to generate various sorts of gene knockout animals, especially of animals in which conventional knockout techniques for rats and mice have not been applicable. The coming decade will be able to see new progress in the functional study of GnRH neurons. However, we should not be only satisfied with the results of such molecular genetic studies but should always bear in mind to get back to the study of physiology of intact animals, whenever it is necessary, to understand the true physiological functions of GnRH neurons. We should also bear in mind not to confine ourselves to the study of model animals but to pay attention also to the evolutionary aspects of the GnRH systems in diverse animal species.

## Conflict of Interest Statement

The authors declare that the research was conducted in the absence of any commercial or financial relationships that could be construed as a potential conflict of interest.
